# Editorial: Managerial decision-making from the perspectives of behavioral science and neuroscience

**DOI:** 10.3389/fpsyg.2023.1125333

**Published:** 2023-01-24

**Authors:** Wuke Zhang, Jie Yu, Liuting Diao, Senqing Qi

**Affiliations:** ^1^Business School, Ningbo University, Ningbo, China; ^2^Nottingham University Business School China, University of Nottingham Ningbo, Ningbo, China; ^3^MOE Key Laboratory of Modern Teaching Technology, Shaanxi Normal University, Xi'an, China

**Keywords:** managerial decision-making, behavioral science, decision neuroscience, emotion, perception, experiences

## Introduction

The past two decades have witnessed the boom of research in exploring managerial decision-making and relevant processes, in which more and more scholars have creatively tried to apply new tools and theories from behavioral science and neuroscience and a series of valuable and remarkable findings have been accomplished in multiple sub-disciplines of management (Camerer and Yoon, 2015; Plassmann et al., 2015; Kirwan et al., 2022; Li et al., 2022). Scholars have used these tools and theories to explore underlying decision-making mechanisms (Ye et al., 2022; Zhang et al., 2022), analyze implicit processes (Plassmann and Weber, 2015; Zhang et al., 2019a), identify individual differences (Plassmann and Weber, 2015; Huang et al., 2021) and produce more accurate predictions of behaviors (Venkatraman et al., 2015; Baldo et al., 2022) in managerial decision-making research areas. Recently, apart from the managerial decision-making periods, researchers are at the same time making more efforts on the fascinating periods preceding and following decision-making behaviors by using behavioral science and neuroscience tools (Karmarkar and Plassmann, 2019).

While acknowledging the development of research in managerial decision-makings, it should also be noticed that most previous studies mainly focused on the research area of marketing especially in consumer behavior, which leaves other managerial decision-making areas, such as investment behavior, operation behavior etc., a void to be filled. Although managerial decisions such as investment and operation are influenced by various factors (e.g., risk, cost, and potential profit), they are finally made by senior executives (Orth, 2002; Zhang et al., 2019b). Thus, tools and theories from behavioral science and neuroscience would also be appliable and accountable for better understanding other managerial decisions mentioned above.

Taking the whole foregoing arguments into consideration, this Research Topic aims to call for a wide range of studies on managerial decision-making using behavioral and/or neuroscience technologies and methods. And it is fortunate to see the Research Topic has attracted a wide range of high-quality submissions from various disciplines. Based on methodologies and research issues, we finally selected 24 exquisite articles in this Research Topic and classified then further into five categories. The main findings and perspectives are summarized as follows.

## Consumer behavior research from the perspective of behavioral science

The first part picks 7 published articles, which mainly adopt empirical methods for consumer behavior research from the behavioral science perspective. With rapid advancement of virtual reality technology, Wang L. et al. investigate the thermal comfort and satisfaction of virtual tourists. Based on the data collected from microclimate simulation experiments and subjects' electrocardiogram, their results show that the thermal comfort of virtual tourists is heavily affected by environmental temperature. Their research provides abundant practical implications, especially in promoting the thermal comfort of tourists and reducing the difference between low and peak travel seasons. Gao, Jiang, et al. explore the source of short video customer inspiration and construct the formation path model of customer inspiration. Their research extends short video marketing and has implications for online marketers. Wang takes a new perspective by focusing on the influence of consumer sentiment on assertive advertisement attitudes. Their research reveals that anxious consumers are more likely to choose assertive advertisement. Ding et al. further expand the existing research on the effects of metaphorical advertisements and straightforward adverts. They explore the effects of both types of advertisements on social media and find that the type of social media advertisement had no significant effect on visual attention. By collecting field data on physiological parameters, Rinklin et al. prove that American and Chinese consumers are different in their exposure to novel products. Their findings suggest that new product development and applied marketing should be adapted to local conditions. Chen T. et al. study the underlying mechanism of how online reviews affect consumer purchasing decisions by using eye-tracking. Their findings suggest that consumers are unable to discern fake reviews and pay more attention to negative ones. Gao, Zeng, et al. prove the effect of presence and customer inspiration on impulse purchase intention in short video marketing by using questionnaire survey and three laboratory experiments. Their results provide valuable reference for marketing strategies to shorten consumers' decision-making time in short video purchase.

## Empirical studies on consumer neuroscience

The second part selects seven papers, which all use neuroscience tools to empirically explore consumer psychology or behavior. Song et al. explore how individuals with different levels of empathy respond to distant brand extensions under corporate social responsibility and corporate competence associations by using ERPs. They provide potential electrophysiological evidence for the positive impact of brand associations on the evaluation of distant brand extension in the case of subdividing different empathic individuals. In an attempt to address the waste of recycled water use on campus, Liu et al. adopt ERPs to explore the effect of social norms on the willingness to use recycled water and the neural mechanism of cognitive processing. They find that college students pay more attention to social norms in groups with closer social distance and thus suggest that forming the social norms of recycled water usage should begin with the group with close social relations. Peng-Li et al. investigate the combined impact of cognitive regulation and ambient noise on food cravings through neurophysiological activity (i.e., electrodermal activity and electroencephalography). Their findings provide a more comprehensive and objective picture of the factors that influence food-related decision-making. With the rapid development of the take-out industry, Wang C. et al. explore how consumers are affected by taste and hygiene ratings by incorporating behavioral and neural approaches. By focusing attention, cognitive conflict, and decisional confidence that are measured by ERPs, their results uncover the decisional process of online food-ordering when consumers are exposed to taste and hygiene ratings simultaneously. Jing et al. adopt ERPs to explore the effectiveness of price promotions in purchasing affordable luxury products and find that price promotions for a high-priced affordable luxury product are effective, but it is not the case for a low-priced affordable luxury product.

In this part, another two more papers used other neuroscience tools, rather than ERPs, to study consumer psychology and behavior. Kim et al. study the neural mechanism of price and customer ratings affecting consumers' purchase of hedonic products by using functional magnetic resonance imaging (fMRI). Their results suggest that brain regions associated with social cognition are involved in customer ratings, not price, in the process of product selection and evaluation. Fu et al. investigate the influence of consumers' implicit awareness on public service announcements by employing functional near-infrared spectroscopy (fNIRs) and find a correlation between activation of dorsolateral prefrontal cortex (dlPFC) and the effectiveness of public service announcements. Their findings suggest that neuroimaging tool (e.g., fNIRs) can also be used to investigate the effectiveness of public service announcements, not just commercial advertisements.

## Business decisions beyond consumer decision-making

In the third part, there are four articles focusing on the business decisions beyond consumer decision-making and various research methods are applied in these articles. Du et al. study the effect of adopting a dual channel supply chain on the performance of a two-level system (manufacturer-retailer) by using a novelty quantitative approach. Their results suggest that the potential market demand for customization affects the price of customized products and the profits of customized channels. From the perspectives of manager cognition and behavior selection, Han et al. discuss the driving mechanism for manufacturer's decision of green innovation. The authors suggest that making green innovation decisions should match the cognition of managers' efficiency logic and their cognition about sustainable development. Wang X. et al. discuss how bank credit willingness is affected by the scale of third-party logistics guaranteeing firms in supply chain finance. They find large-scale 3PL guaranteeing firms receive more positive comments from credit decision makers. Their study reveals the neural processing of credit decisions and expands the theory of credit scale discrimination in the field of decision neuroscience, which is quite rare in previous studies. To address the problem of frequent dishonest transactions by online shopping platform merchants, Chen H. et al. develop monopoly and competitive platform pricing models based on two-sided market theory. They find the impacts of monopoly and consumer information levels on platform pricing, number of bilateral users, and profits.

## Basic studies in the field of management psychology

The fourth part contains four papers that explore contemporarily valuable and fundamental issues in the field of management psychology. Zhou et al. explore how incidental affect impact on intertemporal choice. Their results of the two studies indicate that positive incidental affect leads to longer time perception and more attention to the delay attribute of intertemporal choice, which leads individuals to prefer immediate options in the intertemporal choice. Boredom is a common emotion suffered by humans, and Chen and Rau study the relationship between boredom and prospective memory. They use alpha oscillations to examine the relationship between the two factors and indicate the key role of attention management and visual information because they could help prepare for prospective memory. Their findings suggest boredom and prospective memory are linked by parietooccipital oscillations. Sugawara and Katahira explore why people persistently pursue a difficult target. They conduct an online experiment to investigate the hypothesis that choice perseverance leads individuals to repeatedly choose a hard-to-get target. An important finding of this article is that people with high choice perseverance pursue hard-to-get targets. Their results are of great significance to understand the psychological mechanism by which people adhere to long-term goals. Zheng et al. study the relationship between response (in)consistency and the first mover's anticipation. They design a dual-player gambling task to investigate how this inconsistency would influence their anticipation. Their results reveal evaluation of the performance feedback in gambles and suggest the consistency in social information affected the anticipation of outcomes.

## Systematic reviews on the research trends of neuroscience in marketing and information system

The fifth part collects two review papers, which analyze the application of neuroscience in the fields of marketing and information system respectively. Zhu et al. conduct a systematic review with a bibliometric analysis on neuromarketing research trend from 2010 to 2021 based on the Web of Science database. The authors explore the mapping of co-citation, bibliographic coupling, and co-occurrence, as well as popular research at different time stages and the research trends of neuromarketing research methods and tools. This study provides an overview of the trends and paths in neuromarketing. As NeuroIS has emerged as a new cutting-edge research field, Lin et al. perform a bibliometric analysis to identify, summarize, and classify existing NeuroIS publications from 2010 to 2021. Their results provide a picture of the development trajectory of NeuroIS studies and reveal potential Research Topics in the future.

## Conclusion

In summary, our Research Topic (RT) distills 24 articles that come from various subjects, covering a wide range of research themes. Based on methodologies and research issues, these articles are classified into five sections. The first section, entitled “Consumer behavior research from the perspective of behavioral science,” shows the values of behavioral methods in exploring the potential influencing factors and causal relationships in consumer behavior field. The second section, entitled “Empirical studies on consumer neuroscience,” indicates the advantages of neuroscience methods in identifying underlying decision-making mechanisms, measuring cognitive and mental processes, and better understanding individual differences. The third section, entitled “Business decisions beyond consumer decision-making,” shows that, apart from consumer decision-making, other business decisions can also be studied from the perspectives of behavioral science and neuroscience, which can also provide valuable insights for the decision-making mechanism. The fourth section, entitled “Basic studies in the field of management psychology,” discusses some fundamental and interesting issues (e.g., intertemporal choice, reasons of pursuing hard-to-get targets) in the field of management psychology by using various behavioral and neuroscience methods. The fifth section, entitled “Systematic reviews on the research trends of neuroscience in marketing and information system,” provides a more holistic picture of current trends and future directions about the application of neuroscience in the fields of marketing and information system.

In the future, we hope to see more international cooperation to generate more up-to-date articles, which can bring us the most cutting-edge managerial decision-making research. More research is also needed to explore different possibilities for the managerial decision-making. At the same time, new research methods and mixed research methods can also be explored in a more creative way to bring us new ideas and insights in our research area. We all sincerely expect that people can learn about interesting knowledge and research of managerial decision-making in our topics.

## Author contributions

WZ and JY wrote the whole manuscript. LD collected the references and all published paper in this Research Topic. SQ provided guidance throughout the entire paper and revised the manuscript. All authors listed have made a substantial, direct, and intellectual contribution to the work and approved it for publication.
